# Decompressive Cholecystocentesis in 14 Dogs with Gallbladder Distension and Persistent Primary Digestive Symptoms

**DOI:** 10.3390/ani16193029

**Published:** 2026-09-26

**Authors:** Jarod E. Williams

**Affiliations:** Ozark Veterinary Specialty Care, Springdale, AR 72762, USA; jarod.williams@thrivepet.com

**Keywords:** gallbladder distension, cholecystocentesis, diurnal inappetence, bilious vomiting

## Abstract

Decompressive cholecystocentesis (DCC) was performed in 14 dogs with gallbladder distension (GBD) and primary digestive symptoms non-responsive to conventional symptomatic therapy. All dogs in this uncontrolled, retrospective case series had their fasting estimated gallbladder volume (eGBV) measured by the ellipsoid method. Aspirated volumes were compared to predicted volumes. Common symptomatic characteristics included diurnal inappetence (DI) and bilious vomiting (BV). Total magnesium insufficiency was common in the general population. In all dogs treated, symptom relief was reported within 24–48 h. Follow up status and data were available for all dogs within a 2–22 month period.

## Simple Summary

Gallbladder enlargement can result in abdominal pain and digestive distress. This small study describes how decompressive aspiration of the enlarged gallbladder may improve persistent digestive symptoms in dogs. In total, 14 dogs were evaluated for ongoing vomiting, diarrhea, and abdominal pain non-responsive to routine medical care. All dogs had gallbladder enlargement, and all had aspiration performed to mitigate their symptoms. All dogs showed symptomatic improvement. Little is known about how gallbladder enlargement affects dogs and how gallbladder volume should be interpreted. We propose pathophysiology and open discussion for future study of this subject.

## 1. Introduction

The technique and safety of diagnostic cholecystocentesis (CC) have been described previously in veterinary medicine [1,2,3]. DCC has been used to relieve biliary obstructive pressure in pancreatic extra-hepatic bile duct obstruction (EHBDO) [4]. Gallbladder volume assessment has also been studied with fasting and post-prandial normal ranges reported in normal dogs. Normal fasting volumes are reported to be around 1 mL/kg and post-prandial volumes reduced by 25–50% of the fasting volume [5,6,7]. Dogs with gallbladder disease, notably with biliary sludge presence and Gallbladder Mucocele (GBM), have been reported to have diminished ejection fraction [8]. A single case series documented altered appetite in relation to decreased gallbladder motility in dogs [9]. Little is known about the relationship between gallbladder volume and primary digestive symptom management in dogs. This case series describes the characteristics and outcomes of DCC as part of symptom management in dogs with ongoing primary digestive symptoms.

## 2. Materials and Methods

A retrospective case series study was evaluated. Given the lack of procedural precedent, informed owner consent was obtained for this non-standard procedure, and the risks and potential benefits were thoroughly discussed prior to intervention. Dogs were included with sonographic GBD (approximately 1 mL/kg or greater estimated by the ellipsoid calculation) and varied but persistent primary digestive symptoms following routine medical management. GBD is specifically phrased to include both the upper limit of normal fasting volume and a pathologic voluminous state without specifying either. EGBV was calculated by the ellipsoid method (0.52(L × W × H)) and compared to collected volumes. Cholecystocentesis was performed under general anesthesia by ultrasound guidance. All bile samples were submitted for infectious disease testing by next generation sequencing. All patients were monitored in hospital for post-procedure complications by routine vital parameters and digestive functional assessment. Effusion evaluation was planned for any dog with fever, abdominal distension, abdominal pain or overall disease persistence. All patients received at least one post-procedure evaluation; fasting eGBV was calculated and compared to initial values. The resolution or persistence of symptoms was analyzed.

## 3. Results

Population characteristics revealed the dogs were aged 6–72 months with a mean of 35 mo. Body weight ranged from 2.3 to 37 kg with a mean of 8.1 kg. Sex distribution was 7/14 FS, 6/14 MN, and 1/14 M. Breed distribution revealed three Daschunds, two Shi Tzu, and two Yorkies, with single various additional breeds.

Presentation clinical characteristics revealed the dogs were hospitalized for 1–3 days duration with varied digestive symptoms not responding to routine triage medical support. Gallbladder distension was defined as approximately 1 mL/kg or greater in 13/14 patients. A single patient’s eGBV was calculated at 0.95 mL/kg. eGBVmean in the population was 2.2 mL/kg. Table 1. Sonographic inspissation was present in 9/14. No dog was characterized with GBM. All dogs reported variations of primary digestive disease including anorexia (12/14), vomiting (11/14), diarrhea (10/14), the presence of DI patterns (7/14) and BV (12/14). Biochemical panel testing varied among cases based on triage clinician preference; reported abnormalities included hepatic enzymopathy; ALT (>125 IU/L, 2/13), ALP (>212 U/L, 2/14), GGT (>11 U/L, 1/6), and tbili (>0.9 mg/dL, 0/7). Leukocytosis was noted; tWBC (>16.76 K/µL, 5/13) and neutrophilia (>11.64 K/µL, 7/13). Hypoalbuminemia (<2.3 g/dL) was not reported (0/14) (IDEXX Labs, Westbrook, ME, USA, https://www.idexx.com). D3 insufficiency (<100 ng/mL) was noted (6/14) with a mean of 97.1 ng/dL (range 24.5–147.6 ng/mL). B12 insufficiency was noted (<300 pg/mL, 2/12). Folate was reported to be normal (4.3–21 ng/mL, 10/12) with folate elevations (2/12). Total magnesium insufficiency was noted (<2.2 mg/dL, 12/14), with a mean of 1.7 mg/dL (range 1.3–2.3 mg/dL) (VDI Labs, Chatsworth, CA, USA, https://vdilab.com/). The measured mean aspirated volume of bile was 2.4 mL/kg (range 0.78–5.9 mL/kg). Residual eGBV post-DCC appeared negligible with most procedures reduced to visible luminal collapse (see Figure 1). The author concedes that an unreliably quantified volume of bile remained that would increase the actual GBV marginally in each case. Utilizing the ellipsoid calculation, the residual volumes were categorized as <1 mL (7/13), 1–2 mL (5/13) and <5 mL (1/13). All samples were submitted for infection testing by next generation sequencing (MiDog Animal Diagnostics, Tuston, CA, USA, https://www.midogtest.com/). In total, 2/14 cases yielded positive results but with no significant pathogen reported. Bile cytology was not submitted for external clinical pathologic review.

During the post-procedure period, no patient required additional evaluation beyond routine examination through hospitalization discharge. No adverse event was recorded in any patient that required additional hospitalization or resource allocation associated with DCC.

Post-procedural data including at least one follow up examination was available for 14/14 cases. Follow up periods of observation ranged from 2 to 22 months with a mean of 10.5 months. All dogs treated showed resolution of their primary symptoms within 24–48 h and were discharged functionally from hospitalization. A combination of treatments was prescribed for all cases. No case received DCC intervention alone, making a definitive association between intervention and recovery impossible. Common pre- and post-DCC treatments including broad spectrum antibiotics (14/14), maropitant (14/14), prokinetics (9/14), sucralfate (8/14), proton pump inhibitors (12/14), fluid therapy (14/14), parenteral Dexamethasone per practice standard of 0.05–0.1 mg/kg once (10/14), oral prednisone/olone at short-term anti-inflammatory dosing < 0.5 mg/kg/day (2/14) and ursodiol at 10–15 mg/kg/day (14/14) were prescribed. The author re-iterates that all patients in the series had been treated with conventional symptomatic therapies employing various combinations of the aforementioned treatments, prior to interventional DCC. Common but varied bland and low-fat, home-cooked to prescription digestive dietary supportive therapies were prescribed or continued in all patients.

Fasted eGBV mean at follow up was reported to be 1.04 mL/kg with 7/14 dogs remaining under 1 mL/kg. In total, 7/14 cases measured 1 mL/kg or greater. Normal appetite was maintained in 12/14 cases with a resolution of DI patterns in 6/7 cases. BV had resolved in 8/12 symptomatic cases. Notably, of the cases in which anorexic, DI and BV symptoms persisted to any reported degree, the mean eGBV was 1.3 mL/kg (range 0.3–2.2 mL/kg) and in cases with a resolution of symptoms eGBV was reported to be 0.8 mL/kg (range 0.5–2.1 mL/kg). Two cases in the series were referred to surgery due to persisting symptoms; those cases reported eGBV values of 1.8 mL/kg (10 months post-DCC) and 2.2 mL/kg (5 months post-DCC). A single dog remained asymptomatic with an eGBV of 2.1 mL/kg (22 months post-DCC). Sonographic inspissation was persistent in 9/14 at follow up. Progression to overt GBM was not noted within the series time frame.

## 4. Discussion

In this population, pre-DCC hospitalization with symptom persistence ranged from 1 to 3 days with a case variable but significant polypharmacy prescribed. Duration of hospitalization could not be factored as an indicator of disease severity due to variations in owner motivation to pursue diagnostic ultrasound. Duration of post-DCC evaluation varied between 2 and 22 months, which is a significant variance. Mean post-DCC evaluation was 10 months. A prolonged and uniform duration of monitoring data would be needed for more definitive assessment. A standardized disease scoring assessment (CIBDAI (Canine Inflammatory Bowel Disease Activity Index) or CCECAI (Canine Chronic Enteropathy Clinical Activity Index)) was not pursued due to the sample size, lack of uniform clinical assessment during triage phase and pre-existing polypharmaceutical interventions. Further controlled evaluation of this population would benefit from more specific categorization.

GBD defined at approximately 1 mL/kg was positively correlated with variable digestive symptoms (anorexia, vomiting, diarrhea, lethargy). Historical and/or chronic symptoms of DI and BV were common in this case population, suggesting an acute on chronic disease element. DI has been reported to be associated with non-gravity-dependent biliary sludge in dogs and improves following cholecystectomy [10]. GBV was not reported in that case series but dogs with biliary sludge are known to have reduced GBEF implying a dyskinetic pathology [8]. Bilious vomiting syndrome (BVS) has been studied and surmised to involve abnormal gastrointestinal motility [11]. Potential cholecystopathic dynamics have not been evaluated in dogs with BV to this author’s knowledge. Cases in this series did not undergo ejection fraction testing, primarily due to their shared functional digestive instability. Ejection fractional testing would be helpful to better understand pathophysiologic dynamics in similar cases. In this case series, varied primary gastrointestinal symptoms (anorexia, vomiting and diarrhea) along with DI and BV patterns responded to combined continued routine medical supportive care (all were under treatment with persisting symptoms) and DCC. The author would propose that GBD may simply be a cause of visceral pain that may improve with DCC when routine medical support has failed. Several cases in this series showed marked GBD > 2–5 mL/kg, not likely to be of acute distension origin, without hyperbilirubinemia or sonographic evidence of extra-hepatic bile duct obstruction. Considering the normal post-prandial physiology of coordinated bile expulsion, food ingestion and gastric distension result in cholecystokinin (CCK) release which stimulates gallbladder contraction and intestinal motility coordinated with gastric emptying. Primary bile acids entering the small intestine are eventually reabsorbed in the ileum, returned to the liver via enterohepatic recirculation, and in turn inhibit further CCK release and end the digestive cycle [12]. In this series, persistent bilious vomiting/reflux was a common symptomatic characteristic in conjunction with GBD (12/14). The author proposes that excessive bile secretion into an empty intestine would predictably favor progressive cholecystostasis, GBD and dysmotility via prolonged CCK inhibition. In turn GBD would favor persistent uncoordinated bile expulsion into the small intestine, especially under the abdominal compartmental compressive dynamics associated with vomiting, likely creating a cyclic pathology. Breaking this cycle with DCC may allow for a pathologic CCK inhibitory intermission if present and may in part explain the symptom relief observed in this population. This is likely an overly simplistic pathophysiology but potentially important. The proposed pathophysiologic dynamics would reasonably apply to both an abrupt distension to normal maximum as well as a chronic pathology with progressive distension to supranormal volumes. Cholecystic dynamics (volume and contractility) in dogs with bile reflux and BVS may benefit from additional study. Through the follow up period, few cases in this series had relapsed or continued symptoms (4/14). In the population studied, fasted pre-DCC mean eGBV was 2.2 mL/kg with the fasted post-DCC mean eGBV of 1.0 mL/kg suggesting improved volume dynamics overall. Two cases were referred for a cholecystectomy due to ongoing symptoms. These two cases, assessed at 5 and 10 months post-DCC, showed persistent GBD at 2.2 and 1.8 mL/kg respectively. The trend noted suggested that persistent GBD may be associated with ongoing symptoms. The EGBV measured in the asymptomatic cases was reported as a mean of 0.8 mL/kg, while the eGBV in the symptomatic cases was reported at a mean of 1.3 mL/kg. However, a single case reported occasional bilious vomiting with an eGBV of 0.3 mL/kg while another case continued to have no symptoms with an eGBV of 2.1 mL/kg which would suggest volume is not the only determining factor.

In this population, magnesium insufficiency defined as total magnesium below 2.2 mg/dL was common (12/14). Mean total magnesium was 1.7 mg/d (normal 1.7–2.9 mg/dL, population range 1.3–2.2 mg/dL). Ionized magnesium is the standard for the diagnosis of clinical hypomagnesemia in dogs and is reported commonly in hospitalized animals [13]. Total magnesium has been shown to correlate with ionized magnesium levels but may be weaker in the hospitalized animal [14]. Fractionation of magnesium occurs as ionized 63%, complexed 6% and protein bound 31% forms [15]. No dog in this series was reported to have hypoalbuminemia, making a hypoproteinemic artifact less likely. Ionized hypocalcemia (IHC) was reported to be a risk factor for ionized hypomagnesemia in hospitalized dogs, most commonly in dogs with gastrointestinal illness [16]. Ionized calcium levels were not available in this population. We have no data on magnesium dynamics in dogs with gallbladder disease. However, it has been reported that oral magnesium administration is used to stimulate GB contraction in people and parenteral magnesium has been used to stimulate gallbladder contraction in dogs, both by CCK release stimulated mechanisms [17,18]. The high incidence of magnesium insufficiency in this population may be a dynamic predisposition or co-morbidity that could benefit from further study.

The case number in this small series is insufficient to have any statistical power of correlation relative to volume and persistence of symptoms and may benefit from further study. In this series, the cause of observed GBD was not identified. EHBDO was excluded in all cases based on ultrasound and normal bilirubin levels. An underlying dyskinetic pathology would require ejection fraction testing which was not performed. A non-obstructive, dyskinetic pathophysiology would require further investigation to clarify categorical stimulation deficits (neurohormonal) or potential smooth muscle contractility deficits (such as electrolyte insufficiencies).

## 5. Conclusions

In this patient population, DCC for GB decompression was performed successfully. GBD was observed with the persistence of digestive symptoms beyond common symptomatic therapies, particularly diurnal inappetence and bilious vomiting. DCC may help in cases with ongoing symptoms and concurrent GBD. DCC did appear to have a reductive effect on follow up fasted eGBV across the series. Next generation sequencing performed in this small population failed to identify a primary infectious element. Objective assessment of symptomatic cholecystopathy in dogs may benefit from characteristics beyond inspissative observations and include volumetric dynamics. Further controlled and thoughtful study is needed.

## Figures and Tables

**Figure 1:**
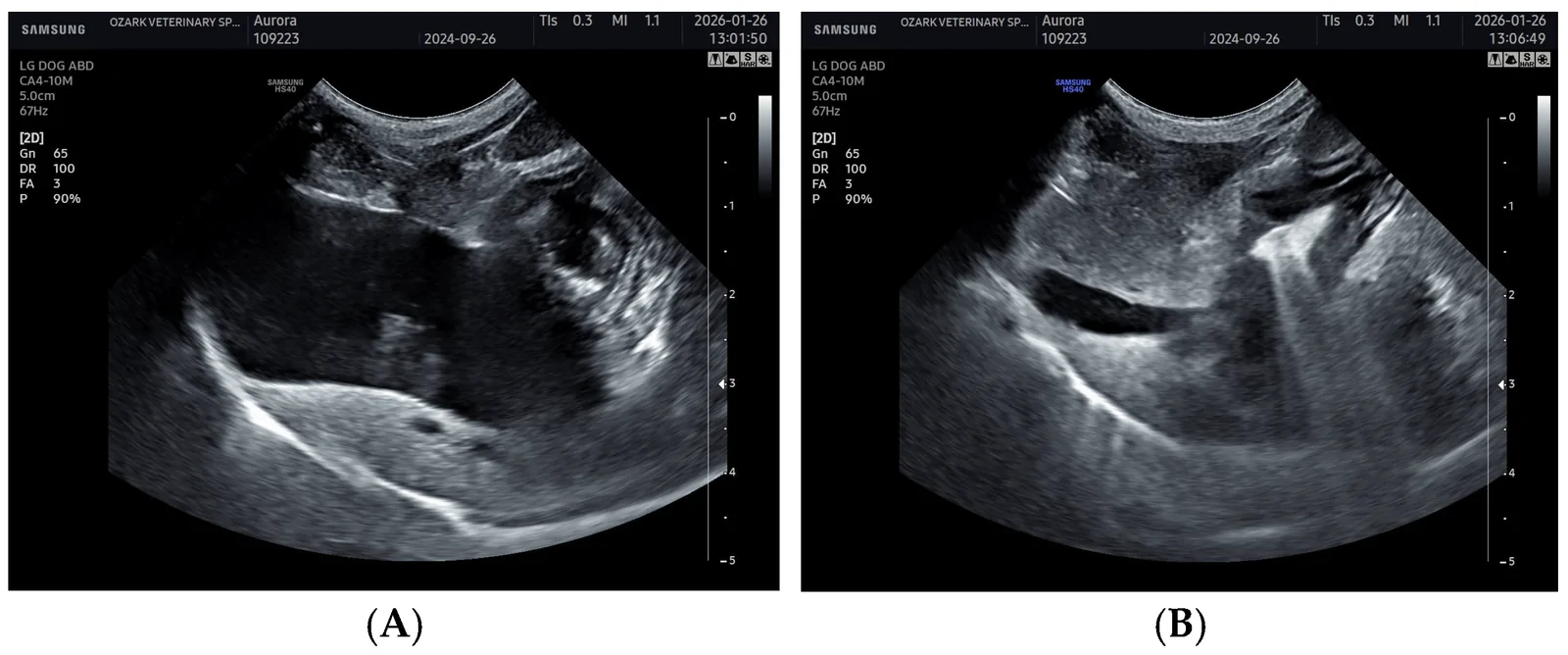
Case 1: Sonography GB long axis view pre- and post-DCC. (**A**) Case 1: pre-DCC, eGBV 5.2 mL/kg; (**B**) Case 1: post-DCC, cBV 4.3 mL/kg, rGBV < 2 mL.

**Table 1:** Decompressive cholecystocentesis dynamic data and timeline.

Case	eGBV (Pre) mL/kg	Inspissate Present	cGBV mL/kg	rGBV	eGBV (Post) mL/kg	Inspissate Present	Time Months	Symptoms
1	5.2	Yes	4.3	<2	2.1	Yes	22	N
2	2.4	No	2.4	<2	0.3	No	4	Yes
3	5.8	No	5.9	<2	1.3	Yes	18	N
4	1.6	Yes	1.7	<2	1	Yes	18	Yes
5	1.2	Yes	1.3	NA	0.5	No	6	N
6	1.5	Yes	2.5	<2	0.6	Yes	12	N
7	0.95	No	0.78	<5	0.8	No	12	N
8	1.7	Yes	1.6	<1	1.8	Yes	10	Yes
9	1.25	No	1.6	<1	0.9	Yes	7	N
10	2.1	Yes	3.6	<1	0.48	No	17	N
11	1.7	No	2.5	<1	0.6	No	4	N
12	2.2	Yes	2.2	<1	2.2	Yes	5	Yes
13	1	Yes	1.4	<1	1	Yes	2	N
14	2.5	Yes	2.5	<1	1	Yes	10	N
Mean	2.22	9/14	2.44		1.04	9/14	10.5	4/14
Median	1.7		2.3		0.95		10	

eGBV: Fasting estimated GBV by ellipsoid equation. cGBV: DCC collected bile volume. rGBV: Estimated residual GBV post-DCC. Case 5 rGBV reported as NA; no rGBV cross-sectional image available for eGBV approximation.

## Data Availability

The original contributions presented in this study are included in the article. Further inquiries can be directed at the corresponding author.

## Abbreviations

The following abbreviations are used in this manuscript:

DCC	Decompressive Cholecystocentesis
GBD	Gallbladder Distension
eGBV	Estimated Gallbladder Volume by ellipsoid calculation
CC	Cholecystocentesis
BV	Bilious Vomiting
DI	Diurnal Inappetence
GBM	Gallbladder Mucocele
EHBDO	Extra-hepatic Bile Duct Obstruction
cBV	Collected Bile Volume
rGBV	Residual Bile Volume by ellipsoid calculation
CCK	Cholecystokinin
GBEF	Gallbladder Ejection Fraction
IHC	Ionized Hypocalcemia
